# A public data set of walking full-body kinematics and kinetics in individuals with Parkinson’s disease

**DOI:** 10.3389/fnins.2023.992585

**Published:** 2023-02-16

**Authors:** Thiago Kenzo Fujioka Shida, Thaisy Moraes Costa, Claudia Eunice Neves de Oliveira, Renata de Castro Treza, Sandy Mikie Hondo, Emanuele Los Angeles, Claudionor Bernardo, Luana dos Santos de Oliveira, Margarete de Jesus Carvalho, Daniel Boari Coelho

**Affiliations:** ^1^Biomedical Engineering, Federal University of ABC, São Bernardo do Campo, Brazil; ^2^Center for Mathematics, Computation, and Cognition, Federal University of ABC, São Bernardo do Campo, Brazil; ^3^Faculdade de Medicina do ABC, Santo André, Brazil

**Keywords:** gait, kinesiology, biomechanics, motor control, movement disorders

## Abstract

**Background:**

To our knowledge, there is no Parkinson’s disease (PD) gait biomechanics data sets available to the public.

**Objective:**

This study aimed to create a public data set of 26 idiopathic individuals with PD who walked overground on ON and OFF medication.

**Materials and methods:**

Their upper extremity, trunk, lower extremity, and pelvis kinematics were measured using a three-dimensional motion-capture system (Raptor-4; Motion Analysis). The external forces were collected using force plates. The results include raw and processed kinematic and kinetic data in c3d and ASCII files in different file formats. In addition, a metadata file containing demographic, anthropometric, and clinical data is provided. The following clinical scales were employed: Unified Parkinson’s disease rating scale motor aspects of experiences of daily living and motor score, Hoehn & Yahr, New Freezing of Gait Questionnaire, Montreal Cognitive Assessment, Mini Balance Evaluation Systems Tests, Fall Efficacy Scale-International–FES-I, Stroop test, and Trail Making Test A and B.

**Results:**

All data are available at Figshare (https://figshare.com/articles/dataset/A_dataset_of_overground_walking_full-body_kinematics_and_kinetics_in_individuals_with_Parkinson_s_disease/14896881).

**Conclusion:**

This is the first public data set containing a three-dimensional full-body gait analysis of individuals with PD under the ON and OFF medication. It is expected to contribute so that different research groups worldwide have access to reference data and a better understanding of the effects of medication on gait.

## Introduction

Several studies seek to understand the gait kinematics of Parkinson’s disease (PD) patients to identify which parameters are altered with the disease. Compared to healthy individuals in general, it is known that these patients tend to present changes in the spatiotemporal parameters of gait, such as a decrease in step length, reduction in gait speed, and difficulty in the start and stop movement (Brognara et al., 2019; Hobert et al., 2019). However, gait is partially responsive to antiparkinsonian medication. Studies show that some spatiotemporal parameters, such as speed and step size, are different when comparing conditions without (OFF) and with medication (ON), while other gait parameters, such as cadence, do not change with the use of the medication (O’Sullivan et al., 1998; Bryant et al., 2011a,b). Most of these studies describe the kinematic parameters related to spatiotemporal characteristics and the ankle range of motion (Morris et al., 1999; Sofuwa et al., 2005). A lack of studies comparing spatiotemporal, kinematic, and kinetic data in a full-body analysis using a three-dimensional (3D) motion-capture system and force plates.

A few gait datasets in PD are available in the literature (Serrao et al., 2018; Gilmore et al., 2019; Li, 2021; de Oliveira et al., 2022; Ribeiro De Souza et al., 2022). Although these datasets are valuable for a wide range of applications, their usefulness is lessened because they are usually limited to a single type of data (e.g., the center of pressure or acceleration data). To address these limitations, this study aimed to create a public data set of 3D overground walking kinematics and kinetics data on individuals with PD in ON and OFF medication.

## Materials and methods

The data collection was performed in the Laboratory of Biomechanics and Motor Control at the Federal University of ABC, Brazil. The local Ethics Committee approved this study (protocol number 21948619.6.0000.5594), and all patients signed a consent form before collecting data. The patients were on a stable dose of l-DOPA for at least 1 month. The idiopathic individuals with PD participated in two experimental sessions for 1 week, one in the ON condition of the medication and the other in the OFF condition. To be considered ON condition, participants had taken dopaminergic medication 1 h before starting the session to ensure dose stabilization. In the OFF condition, the participants spent at least 12 h without medication for Parkinson’s disease (Araujo-Silva et al., 2022). The order of the sessions was randomized among the patients. The start time of the experiment was the same as the experimental sessions.

### Participants

A convenience sample of 26 idiopathic individuals with PD (6 females and 20 males) was recruited to participate in this study. The patients were recruited from local communities and included local neighborhoods and ambulatory movement disorders. The patients were interviewed to collect information about their demographic characteristics, socio-cultural characteristics, and overall health condition. Their ages varied from 44 to 81 years, body masses from 53.3 to 94.6 kg, heights from 151.5 to 179.0 cm, body-mass indexes (BMI) from 19.2 to 34.3 kg/m^2^, Hoehn & Yahr (H&Y) scale between 1 and 4, and 13 with freezing of gait (FoG). Inclusion criteria were the absence of neurological or physical dysfunctions other than those associated with PD and no diagnosed vestibular, visual, or somatosensory dysfunctions as self-declared.

### Procedures

All gait trials were performed barefoot, and the participants wore comfortable shorts (women wore sports bras). Participants were asked to perform overground walking trials at a self-selected comfortable speed. The marker-set protocol adopted for this study comprised: (a) in the lower limb, 26 anatomical reflective markers (Leardini et al., 2007); and (b) trunk and upper limb, 18 anatomical reflective markers following the recommendations of the International Society of Biomechanics (Wu et al., 2002, 2005); totaling 44 anatomical reflective markers (see Supplementary material for the anatomical description of reflective markers). The following data-collection procedures were implemented.

**FIGURE 1:**
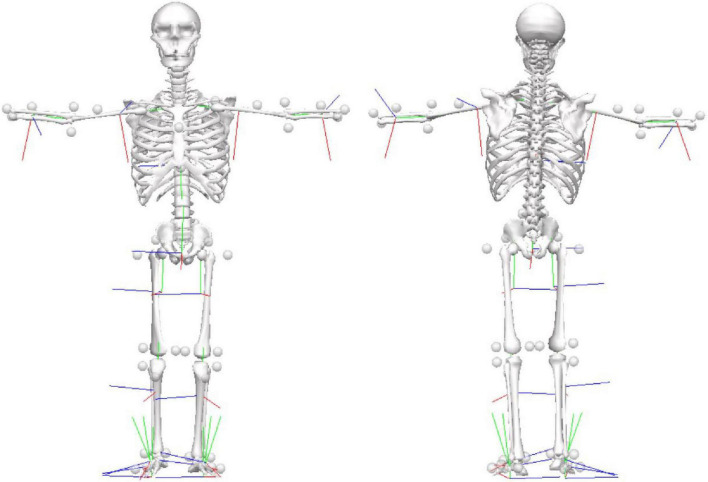
Marker-set protocol. Location of reflective markers for the full-body during the static condition in the anterior and posterior views.

1. The researcher explained to each patient the process of data collection. The patient was informed that he or she would be monitored during the data collection. There should not be any verbal communication during the trials, but he or she could interrupt the data collection if desired. Furthermore, that assistance would be given if necessary.
2. After these explanations, the patient signed the informed consent form.
3. The researcher interviewed the subject to collect information about his or her clinical data, medication, and disease diagnosis.
4. At the beginning of each session, two experienced physiotherapists in movement disorders applied the following scales: Unified Parkinson’s Disease Rating Scale motor aspects of experiences of daily living (UPDRS-II) and motor aspects (UPDRS-III) (Fahn and Elton, 1987), H&Y (Hoehn and Yahr, 2001), New Freezing of Gait Questionnaire (NFoG-Q) (Nieuwboer et al., 2009), Montreal Cognitive Assessment–MoCA (Nasreddine et al., 2005), Mini Balance Evaluation Systems Tests (mini-BESTest) (Horak et al., 2009), Fall Efficacy Scale-International–FES-I (Yardley et al., 2005), Stroop test (Stroop, 1935; Scarpina and Tagini, 2017), and Trail Making Test (TMT) A and B (Spreen and Strauss, 1998). The assessments of each item on the scales were given by consensus among researchers.
5. Participants rested for 10 min.
6. Markers were placed directly onto the skin of the full body (Figure 1).
7. A standing anatomical calibration trial was performed with the participant standing still for 1 s, adopting a stationary T-pose and the feet in a standard position parallel to the *X*-axis of the laboratory coordinate system (LCS) (Fukuchi et al., 2018). A template was used to ensure that the long axes of the feet were aligned with the *X*-axis of the LCS.
8. After the calibration trial and a familiarization period, participants were instructed to walk at a comfortable self-selected speed along a 20-m walkway. Participants performed 20 trials. The participants did not use any aid during the trials.

### Data acquisition and processing

Standard gait analysis was collected using a motion-capture system that had 12 cameras (Raptor-4; Motion Analysis Corporation, Santa Rosa, CA, USA), five force platforms (three 40 × 60-cm Optima models; AMTI, Watertown, MA, USA; two 40 × 60-cm 9281EA models; Kistler, Winterthur, Switzerland) embedded in the floor. The kinematic data were acquired at 150 Hz, and the data on ground-reaction forces were acquired at 300 Hz using a motion-capture system (Cortex 6.0; Motion Analysis, Santa Rosa, CA, USA).

The data processing was performed using Cortex 6.0. Visual 3D software version 6.00.33 (C-motion Inc., Germantown, MD, USA) performed all kinematics and kinetics calculations. To enable users to process the data in the Visual 3D software, a Visual 3D pipeline file is available at Figshare. The analysis of the overground trials considered only those files that contained at least one full gait cycle (stance and swing phase) detected by kinematics. Heel strike and toe-off were calculated bilaterally using the horizontal velocity of the heel marker method (Zeni et al., 2008). The public data set consists of raw c3d and ASCII files containing 3D marker coordinates, angular kinematics, and external forces. In addition, time-normalized kinematic and kinetic average curves, which were considered processed data, were calculated for each participant.

## Results

The data is available at Figshare^1^ in both c3d and ASCII file formats. The data set comprises a file with metadata, and separate text files were generated for the markers, angular kinematics, and force signals at 150 Hz. The clinical characteristics of the patients are presented in Tables 1, 2. There were no episodes of freezing of gait.

**TABLE 1:** Mean (standard deviation) of the characteristics of the participants.

	Characteristics
Disease duration (years)	9.46 (5.66)
L-Dopa equivalent units (mg⋅day^–1^)	845.65 (489.75)
NFoG-Q (score)	8.73 (10.10)
H&Y stage (score)	2.28 (0.68)

H&Y, Hoehn & Yahr; NFoG-Q, New Freezing of Gait Questionnaire.

**TABLE 2:** Mean (standard deviation) of the characteristics of the participants separately by medication condition.

	ON	OFF
MoCA (score)	22.80 (4.25)	23.35 (4.06)
UPDRS-II (score)	4.48 (3.56)	6.57 (3.79)
UPDRS-III (score)	24.80 (13.60)	26.43 (11.76)
UPDRS-III rigidity item (score)	5.20 (3.79)	5.17 (3.05)
UPDRS-III gait item (score)	0.64 (0.81)	1.09 (0.79)
Mini-BESTest (score)	25.04 (5.33)	24.30 (5.51)
FES-I (score)	27.72 (10.29)	33.91 (12.59)

MoCA, Montreal Cognitive Assessment; UPDRS-II, motor aspects of experiences of daily living Unified Parkinson’s disease rating scale; UPDRS-III, motor score Unified Parkinson’s disease rating scale (total score and a separate score for items 5–rigidity–and 12–gait); Mini-BESTest, Mini-Test of Balance Assessment System scale; FES-I, Falls Efficacy Scale International.

### Metadata

The metadata file named PDGinfo.txt contains 61 information from each patient’s anamnesis and clinical scales. Here is the coding for the metadata:

1. ID: the file name of the stabilography trial (SUBXX, where SUB means subjects; XX identifies the patient and varies from 01 to 26).
2. Gender: gender (F or M).
3. Age: patient’s age in years.
4. Height (cm): height in meters (measured with a calibrated stadiometer).
5. Weight (kg): weight in kilograms (measured with a calibrated scale).
6. BMI (kg/m^2^): body mass index in kg/m^2^.
7. Ortho-Prosthesis: name of the orthosis or prosthesis the subject wears (“No” if the patient did not wear any orthosis or prosthesis).
8. Years of formal study: years of formal education.
9. Disease duration (years): year from diagnosis.
10. L-Dopa equivalent units (mg⋅day^–1^): total daily *levodopa equivalent* dose in mg⋅day^–1^ according to Tomlinson et al. (2010).
11. FoG group: presence (freezers) or not (non-freezers) of freezing of gait.
12. NFoG-Q (score): score of New Freezing of Gait Questionnaire.
13. Initial symptoms: self-reported initial symptoms.
14. Is there a family history of PD? Who?
15. Do you feel improvement after using the antiparkinsonian medicine?: Yes or No.
16. Have you ever had any surgery? Which?
17. Any rehabilitation or physical activity?: name of the rehabilitation or physical activity performed by the patient (“No” if the patient did not perform any rehabilitation or physical activity).
18. Other diseases (cardiovascular, bone, etc.): name of the disability of the patient (“No” if the patient did not present any disability).
19. Handedness: a self-reported manual preference.
20. ON–Hoehn & Yahr: Hoehn & Yahr score in the ON medication.
21. ON–MoCA: MoCA score in the ON medication.
22. ON–miniBESTest: miniBESTest score in the ON medication.
23. ON–FES-I: FES-I score in the ON medication.
24. ON–UPDRS-II: total score of the UPDRS-II in the ON medication.
25. ON–UPDRS-II–walking: score of item 4–walking of the UPDRS-II in the ON medication.
26. ON–UPDRS-III: total score of the UPDRS-III in the ON medication.
27. ON–UPDRS-III–Rigidity: score of item 5–rigidity of UPDRS-III in the ON medication.
28. ON–UPDRS-III–Gait: score of item 12–rigidity of UPDRS-III in the ON medication.
29. ON–PIGD or TD: Postural Instability/Gait Difficulty (PIGD) or Tremor Dominant (TD) phenotypes in the ON medication, according to Stebbins et al. (2013).
30. ON–UPDRS-III asymmetry: clinical asymmetry was defined as the difference between the summed UPDRS scores of the left and right body sides (items 3.3–3.8 and 3.15–3.17). The most affected body side was the side with the highest UPDRS score in the ON medication.
31. ON–Stroop-I time (s): time to complete part I of the Stroop test in the ON medication.
32. ON–Stroop-I error: number of errors presented in part I of the Stroop test in the ON medication.
33. ON–Stroop-II time (s): time to complete part II of the Stroop test in the ON medication.
34. ON–Stroop-II error: number of errors presented in part II of the Stroop test in the ON medication.
35. ON–Stroop-III time (s): time to complete part III of the Stroop test in the ON medication.
36. ON–Stroop-III error: number of errors presented in part III of the Stroop test in the ON medication.
37. ON–TMTA time (s): time to complete part A of the TMT in the ON medication.
38. ON–TMTA error: number of errors presented in part A of the TMT in the ON medication.
39. ON–TMTB time (s): time to complete part B of the TMT in the ON medication.
40. ON–TMTB error: number of errors presented in part B of the TMT in the ON medication.
41. OFF–Hoehn & Yahr: Hoehn & Yahr score in the OFF medication.
42. OFF–MoCA: MoCA score in the OFF medication.
43. OFF–miniBESTest: miniBESTest score in the OFF medication.
44. OFF–FES-I: FES-I score in the OFF medication.
45. OFF–UPDRS-II: total score of the UPDRS-II in the OFF medication.
46. OFF–UPDRS-II–walking: score of item 4–walking of the UPDRS-II in the OFF medication.
47. OFF–UPDRS-III: total score of the UPDRS-III in the OFF medication.
48. OFF–UPDRS-III–Rigidity: score of item 5–rigidity of UPDRS-III in the OFF medication.
49. OFF–UPDRS-III–Gait: score of item 12–rigidity of UPDRS-III in the OFF medication.
50. OFF–PIGD or TD: Postural Instability/Gait Difficulty (PIGD) or Tremor Dominant (TD) phenotypes in the OFF medication, according to Stebbins et al. (2013).
51. OFF–UPDRS-III asymmetry: clinical asymmetry was defined as the difference between the summed UPDRS scores of the left and right body sides (items 3.3–3.8 and 3.15–3.17). The most affected body side was the side with the highest UPDRS score in the OFF medication.
52. OFF–Stroop-I time (s): time to complete part I of the Stroop test in the OFF medication.
53. OFF–Stroop-I error: number of errors presented in part I of the Stroop test in the OFF medication.
54. OFF–Stroop-II time (s): time to complete part II of the Stroop test in the OFF medication.
55. OFF–Stroop-II error: number of errors presented in part II of the Stroop test in the OFF medication.
56. OFF–Stroop-III time (s): time to complete part III of the Stroop test in the OFF medication.
57. OFF–Stroop-III error: number of errors presented in part III of the Stroop test in the OFF medication.
58. OFF–TMTA time (s): time to complete part A of the TMT in the OFF medication.
59. OFF–TMTA error: number of errors presented in part A of the TMT in the OFF medication.
60. OFF–TMTB time (s): time to complete part B of the TMT in the OFF medication.
61. OFF–TMTB error: number of errors presented in part B of the TMT in the OFF medication.

### Processed data

The C3Dfiles folder contains a folder for each participant and medication condition. For each participant, the following files are available for each trial separately:

1. c3d files (static and gait trials): The c3d files can store both the 3D coordinates of the markers and the force signals in the same file. In addition, the static trial (the standing anatomical calibration trial), which contains only marker trajectories, is available;
2. Visual 3D pipeline file (Parkinson.v3s) to enable users to process the data in the Visual 3D software;
3. Model templates file (Parkinson.mdh) that contains the definitions of all landmarks, segments, and segment properties;
4. Angular kinematics of all frames of the joints of the full body (shoulder, elbow, trunk, pelvis, hip, knee, ankle and foot) on the right and left side;
5. Linear Kinematics of all frames of the coordinates of the markers and center of mass (CoM);
6. GRF of all frames on the right and left side in the anteroposterior, vertical and mediolateral directions;
7. For each gait cycle, spatiotemporal parameters, such as stride and step length and duration, and cadence.

The gait cycles folder provides the average ensemble data for each participant and medication throughout the full gait cycle (101 time-normalized points; the signal was interpolated using cubic spline) of the following data:

1. CoM: center of mass of the body;
2. Files named with ending Ang: angular kinematics of the joints of the full body (shoulder, elbow, trunk, pelvis, hip, knee, ankle, and foot) on the right and left side. Each gait cycle is organized into three columns:
a. Elbow: flexion/extension, add/abduction, and pron/supination;
b. Shoulder: flexion/extension, add/abduction, and int/external rotation;
c. Trunk: lateral flexion, rotation, and flexion/extension;
d. Pelvis: tilt, obliquity, and rotation;
e. Hip: flexion/extension, add/abduction, and int/external rotation;
f. Knee: flexion/extension, add/abduction, and int/external rotation;
g. Ankle: dorsi/plantarflexion, inv/eversion, and add/abduction;
h. Foot: dorsi/plantarflexion, inv/eversion, and int/external rotation.
3. Files named with ending grf contain three tabs (1) mean and (2) standard deviation of all gait cycles of the GRF on the right and left side in the anteroposterior, vertical, and mediolateral directions;
4. Files named with ending kinematics contain three tabs (1) mean and standard deviation of the spatiotemporal parameters; (2) mean of all gait cycles of the angular and linear kinematics on the right and left side; and (3) standard deviation of all gait cycles of the angular and linear kinematics on the right and left side.

### Data exploration

**FIGURE 2:**
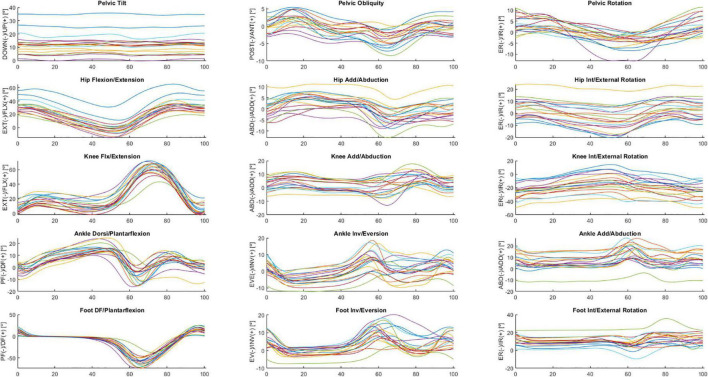
Ensemble averages across participants in ON medication of the pelvic tilt, pelvic obliquity, pelvic rotation, hip flexion/extension, hip add/abduction, hip int/external rotation, knee flx/extension, knee add/abduction, knee int/external rotation, ankle dorsi/plantarflexion, ankle inv/eversion, ankle add/abduction, foot DF/plantarflexion, foot inv/eversion, and foot int/external rotation angles. Each waveform represents a participant.

**FIGURE 3:**
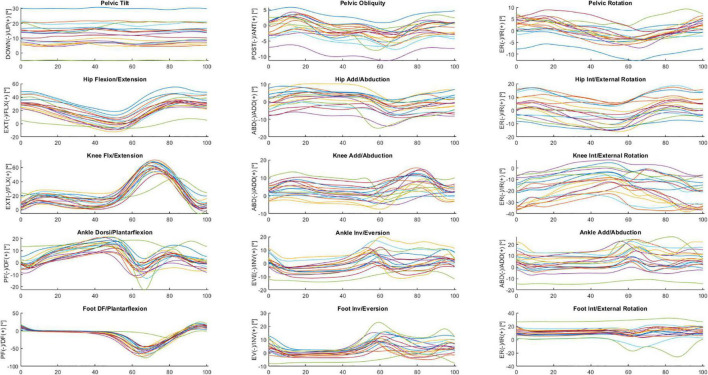
Ensemble averages across participants in OFF medication of the pelvic tilt, pelvic obliquity, pelvic rotation, hip flexion/extension, hip add/abduction, hip int/external rotation, knee flx/extension, knee add/abduction, knee int/external rotation, ankle dorsi/plantarflexion, ankle inv/eversion, ankle add/abduction, foot DF/plantarflexion, foot inv/eversion, and foot int/external rotation angles. Each waveform represents a participant.

The following is a partial exploratory analysis of the data. The curves in this section represent the ensemble average across all participants, only the right leg and the pelvis curves. Figures 2, 3 show the hip, knee, and ankle joint angles and the pelvis and foot segments at the sagittal, frontal, and transverse planes.

**FIGURE 4:**
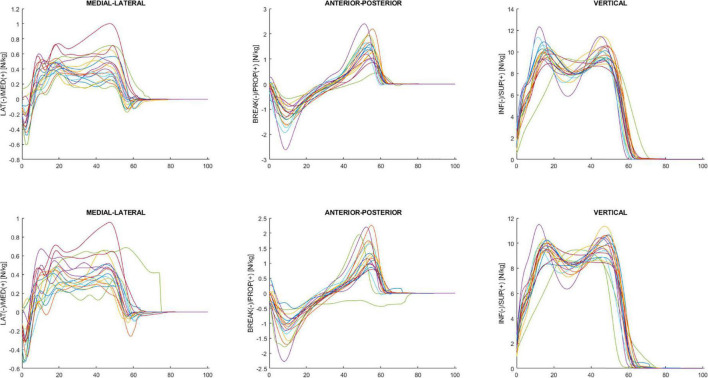
Ensemble averages across participants in ON **(upper panel)** and OFF **(bottom panel)** medication of the mediolateral, anterior-posterior, and vertical ground reaction force. Each waveform represents a participant.

Figure 4 shows GRF data for the mediolateral, anterior-posterior, and vertical direction during the overground walking in ON (upper panel) and OFF (bottom panel).

## Discussion

This study presents a public data set of overground walking kinematics and kinetics in ON and OFF medication for 26 individuals with PD (DOI:10.6084/m9.figshare.14896881). The study contains raw data comprising marker trajectories and GRFs and processed data comprising joint angles that characterize the gait pattern of each participant. A limitation is that there was no foot contact with the force plate in some trials. This circumstance limits the usability of the force plate data. Our sample is small and heterogeneous. Future collections may complement our data set.

Reduced forward limb propulsion is likely caused by impaired muscular contraction, stiffness, and postural instability (Albani et al., 2014). This might negatively impact spatiotemporal gait metrics such as speed and step length. However, there is only partial evidence regarding the relationship between disease pathology, range of motion, force production, the timing of their onset, and their contribution to impaired gait and decreased function because there is insufficient access to motion analysis technology and a lack of expertise to evaluate these gait characteristics. In addition, treatments were typically generic and not designed for specific phenotypes or illness states, which limited their applicability. While several gait data sets are available, only a few are specific to individuals with PD, including with freezing of gait. This data set may be used to enhance knowledge related to the influence of disease on gait biomechanics.

For example, our data set could contribute to understanding clinical scales’ correlations with spatiotemporal, kinematic, and kinetic variables. Non-motor symptoms can exacerbate slow gait, leading to great variability, and trigger FoG (Gilat et al., 2018). Assessing the interaction of non-motor symptoms with the progression of gait impairments is important to improve disease management and treatment. Additional studies correlating clinical scales with gait are needed.

Individuals with PD have a smaller range of motion of the hips in the coronal plane and pelvic obliquity, lower range of flexion-extension of the knees with a high degree of flexion in the initial contact and the stance phase, and greater ankle dorsiflexion during the stance phase compared to healthy subjects (Morris et al., 2005; Sale et al., 2013; Albani et al., 2014; Pistacchi et al., 2017; Zanardi et al., 2021). Reduced arm swing is present in the early stages of the disease (Schneider et al., 2012), and is a predictor of the occurrence of falls (Wood et al., 2002). Arm swing is especially reduced on the most affected side of the body, but this asymmetry decreases as the disease progresses. However, these studies did not assess the effect of FoG on lower and upper limb kinematic parameters in the gait of individuals with PD.

Specific gait characteristics of PD worsen with the course of the disease. Therefore, an objective and quantitative gait analysis system may enhance the current practice (semiquantitative gait evaluation), which may help with PD diagnosis, symptom monitoring, therapy management, rehabilitation, and fall risk assessment and prevention. In addition, improving Parkinson’s disease individuals’ conditions is a major challenge that can be addressed with new emerging technologies such as collaborative robots to assist them during rehabilitation treatments or wearable sensors and devices to monitor and alert them to fall-risk situations; machine learning techniques can increase the effectiveness of these assistive and signaling devices by giving them some awareness of the patients’ situations.

## Data availability statement

The datasets presented in this study can be found in online repositories. The names of the repository/repositories and accession number(s) can be found below: 10.6084/m9.figshare.14896881.

## Ethics statement

The studies involving human participants were reviewed and approved by Federal University of ABC. The patients/participants provided their written informed consent to participate in this study.

## Author contributions

TS: methodology, data curation, and writing—original draft. TC, CO, RC, SH, EL, CB, and LS: methodology and data curation. MJ: writing—review and editing and supervision. DC: conceptualization, methodology, formal analysis, writing—review and editing, supervision, and project administration. All authors contributed to the article and approved the submitted version.

## Footnotes

1
https://figshare.com/articles/dataset/A_dataset_of_overground_walking_full-body_kinematics_and_kinetics_in_individuals_with_Parkinson_s_disease/14896881
