# Supplementary material for: A public data set of walking full-body kinematics and kinetics in individuals with Parkinson’s disease
Source: Front Neurosci. 2023 Feb 16;17:992585. doi: 10.3389/fnins.2023.992585 (PMC9978741; doi:10.3389/fnins.2023.992585)
Supplement: Supplementary file 1 [file Table_1.docx]

**Table 1.** Details of the 44 anatomical reflective markers used to determine the position and orientation of the body segments during walking trials.

| **Label** | **Name** | **Description** |
| --- | --- | --- |
| R.ASIS | Right Anterior Superior Iliac Spine | Right anterior superior iliac spine |
| L.ASIS | Left Anterior Superior Iliac Spine | Left anterior superior iliac spine |
| R.PSIS | Right Posterior Iliac Spine | Right posterior superior iliac spine |
| L.PSIS | Left Posterior Iliac Spine | Left posterior superior iliac spine |
| R.GTR | Right Greater Trochanter | Most lateral prominence of the right greater trochanter |
| R.Knee | Right Knee | Most lateral prominence of the right lateral femoral epicondyle |
| R.Knee.Medial | Right Knee Medial | Most medial prominence of the right lateral femoral epicondyle |
| R.HF | Right Head of Fibula | Proximal tip of the head of the right fibula |
| R.TT | Right Tibial Tuberosity | Most anterior border of the right tibial tuberosity |
| R.Ankle | Right Ankle | Lateral prominence of the right lateral malleolus |
| R.Ankle.Medial | Right Ankle Medial | Most medial prominence of the right medial malleolus |
| R.Heel | Right Heel Bottom | Aspect of the Achilles tendon insertion on the right calcaneous |
| R.MT1 | Right 1^st^ Metatarsal | Dorsal margin of the right 1^st^ metatarsal head |
| R.MT5 | Right 5^th^ Metatarsal | Dorsal margin of the right 5^th^ metatarsal head |
| R.MT2 | Right 2^nd^ Metatarsal | Dorsal margin of the right 2^nd^ metatarsal head |
| L.GTR | Left Greater Trochanter | Most lateral prominence of the left greater trochanter |
| L.Knee | Left Knee | Most lateral prominence of the left lateral femoral epicondyle |
| L.Knee.Medial | Left Knee Medial | Most medial prominence of the left lateral femoral epicondyle |
| L.HF | Left Head of Fibula | Proximal tip of the head of the left fibula |
| L.TT | Left Tibial Tuberosity | Most anterior border of the left tibial tuberosity |
| L.Ankle | Left Ankle | Lateral prominence of the left lateral malleolus |
| L.Ankle.Medial | Left Ankle Medial | Most medial prominence of the left medial malleolus |
| L.Heel | Left Heel Bottom | Aspect of the Achilles tendon insertion on the left calcaneous |
| L.MT1 | Left 1^st^ Metatarsal | Dorsal margin of the left 1^st^ metatarsal head |
| L.MT5 | Left 5^th^ Metatarsal | Dorsal margin of the left 5^th^ metatarsal head |
| L.MT2 | Left 2^nd^ Metatarsal | Dorsal margin of the left 2^nd^ metatarsal head |
| CLAV | Incisura jugularis | Deepest point of Incisura Jugularis (suprasternal notch) |
| STRN | Processus xiphoideus | Most caudal point on the sternum |
| C7 | Seventh cervical vertebra | Processus spinosus (spinous process) of the 7th cervical vertebra |
| T10 | Tenth thoracic vertebra | Processus spinosus (spinal process)of the 10th thoracic vertebra |
| RSHO | Right shoulder | Most dorsal point on the acromioclavicular joint (shared with the scapula) |
| RUPA | Right upper arm | Between the elbow and the shoulder markers |
| REL | Right lateral epicondyle | Most caudal point on lateral epicondyle |
| REM | Right medial epicondyle | Most caudal point on medial epicondyle |
| RFRA | Right lower arm | Between the elbow and the wrist markers |
| RWL | Right lateral wrist | Most caudal–lateral point on the radial styloid |
| RWM | Right medial wrist | Most caudal–medial point on the ulnar styloid |
| LSHO | Left shoulder | Most dorsal point on the acromioclavicular joint (shared with the scapula) |
| LUPA | Left upper arm | Between the elbow and the shoulder markers |
| LEL | Left lateral epicondyle | Most caudal point on lateral epicondyle |
| LEM | Left medial epicondyle | Most caudal point on medial epicondyle |
| LFRA | Left lower arm | Between the elbow and the wrist markers |
| LWL | Left lateral wrist | Most caudal–lateral point on the radial styloid |
| LWM | Left medial wrist | Most caudal–medial point on the ulnar styloid |
